# Nurse-led advance care planning with older people who have end-stage kidney disease: feasibility of a deferred entry randomised controlled trial incorporating an economic evaluation and mixed methods process evaluation (ACReDiT)

**DOI:** 10.1186/s12882-020-02129-5

**Published:** 2020-11-13

**Authors:** Peter O’Halloran, Helen Noble, Kelly Norwood, Peter Maxwell, Fliss Murtagh, Joanne Shields, Robert Mullan, Michael Matthews, Christopher Cardwell, Mike Clarke, Rachael Morton, Karan Shah, Trisha Forbes, Kevin Brazil

**Affiliations:** 1 School of Nursing and Midwifery, Queen’s University Belfast, Medical Biology Centre, 97 Lisburn Road, Belfast, BT9 7BL UK; 2 School of Psychology, Ulster University, Cromore Road, Coleraine, Co. Londonderry, BT52 1SA UK; 3 School of Medicine, Dentistry and Biomedical Sciences, Queen’s University Belfast, Whitla Medical Building, 97 Lisburn Road, Belfast, BT9 7BL UK; 4 Regional Nephrology Unit, Belfast City Hospital, 51 Lisburn Road, Belfast, BT9 7AB UK; 5 Hull York Medical School, University of Hull, Allam Medical Building, Hull, HU6 7RX UK; 6 Renal Unit, Antrim Area Hospital, Bush Road, Antrim, BT41 2RL UK; 7 Centre for Public Health, Queen’s University Belfast, Institute of Clinical Sciences, Royal Victoria Hospital, Belfast, BT12 6BA UK; 8 NHMRC Clinical Trials Centre, University of Sydney, Medical Foundation Building, 92-94 Parramatta Rd, Camperdown, NSW 2050 Australia

**Keywords:** Advance care planning, Advance directives, End-of-life care, End-stage Kidney disease, Feasibility studies, Palliative care, Randomized controlled trial, Renal Dialysis

## Abstract

**Background:**

Advance Care Planning is recommended for people with end-stage kidney disease but evidence is limited. Robust clinical trials are needed to investigate the impact of advance care planning in this population. There is little available data on cost-effectiveness to guide decision makers in allocating resources for advance care planning. Therefore we sought to determine the feasibility of a randomised controlled trial and to test methods for assessing cost-effectiveness.

**Methods:**

A deferred entry, randomised controlled feasibility trial, incorporating economic and process evaluations, with people with end-stage kidney disease, aged 65 years or older, receiving haemodialysis, in two renal haemodialysis units in Northern Ireland, UK. A nurse facilitator helped the patient make an advance care plan identifying: a surrogate decision-maker; what the participant would like to happen in the future; any advance decision to refuse treatment; preferred place of care at end-of-life.

**Results:**

Recruitment lasted 189 days; intervention and data collection 443 days. Of the 67 patients invited to participate 30 (45%) declined and 36 were randomised to immediate or deferred advance care plan groups. Twenty-two (61%) made an advance care plan and completed data collection at 12 weeks; 17 (47.2%) were able to identify a surrogate willing to be named in the advance care plan document. The intervention was well-received and encouraged end-of-life conversations, but did not succeed in helping patients to fully clarify their values or consider specific treatment choices. There was no significant difference in health system costs between the immediate and deferred groups.

**Conclusions:**

A trial of advance care planning with participants receiving haemodialysis is feasible and acceptable to patients, but challenging. A full trial would require a pool of potential participants five times larger than the number required to complete data collection at 3 months. Widening eligibility criteria to include younger (under 65 years of age) and less frail patients, together with special efforts to engage and retain surrogates may improve recruitment and retention. Traditional advance care planning outcomes may need to be supplemented with those that are defined by patients, helping them to participate with clinicians in making medical decisions.

**Trial registration:**

Registered December 16, 2015. ClinicalTrials.gov Identifier: NCT02631200.

## Background

In 2017, more than 25,000 individuals in the United Kingdom (UK) were receiving haemodialysis for end-stage kidney disease (ESKD) [1]. This is mirrored worldwide, with over 2.5 million people receiving renal replacement therapy [2]. Patients with ESKD characteristically suffer from a wide range of co-morbidities, including hypertension, diabetes, heart failure, chronic pulmonary disease, atrial fibrillation, depression, and dementia [3]. Consequently, they are at high risk for hospitalisation [4, 5] and earlier death [6, 7]. Nevertheless, many patients with ESKD do not discuss issues such as cardiopulmonary resuscitation, Intensive Care Unit admission, withdrawal of dialysis, involvement of specialist palliative care, end-of-life care, and preferred place of death with healthcare professionals [8, 9]. Many persons with ESKD also have unmet palliative care needs [10, 11]. In this context, advance care planning (ACP) may enable shared decision-making among patients, their families and healthcare professionals.

ACP is a process of discussions between a patient, their family and healthcare professionals to clarify values, treatment preferences and goals of end-of-life care [12]. In a variety of settings, ACP is associated with important benefits, including reduced hospitalisations increased uptake of palliative care services, improved quality of life, decreased anxiety and depression among family members, and care that is more in keeping with patient preferences [13–17]. Consequently, ACP is widely recommended for patients with ESKD [18–20]. However, recent systematic reviews found sparse, low-quality data to support the benefits of ACP in this population [12, 21]. Robust clinical trials are needed to investigate the impact of ACP on patient and surrogate decision-making and emotional burden; enactment of patient preferences for end-of-life care; recourse to life-prolonging treatments; and use of palliative care services and hospice care. There is also little available data on cost-effectiveness to guide decision makers in allocating resources for ACP [22]. Given that implementation success is heavily influenced by a complex network of patient, staff, organisational and cultural factors [23], and following guidance from the UK Medical Research Council on the evaluation of complex interventions [24], we tested intervention and research processes related to ACP for patients with ESKD. This work will inform a larger scale randomised controlled trial, economic evaluation and process evaluation of ACP for people receiving haemodialysis. We planned deferred entry for the control group, because we wanted to gauge whether patients allocated to this group would be distressed at having to wait for the intervention once the issue of ACP had been raised with them.

### Aim

To determine the feasibility of conducting a deferred entry randomised controlled trial of ACP with patients who have ESKD, incorporating an economic evaluation and mixed methods process evaluation.

Our objectives were to investigate:

- Recruitment, retention and participation rates
- Acceptability of the intervention to patients, their relatives, and healthcare professionals
- Optimal systems for delivering ACP, including the recruitment and training of clinical staff to facilitate ACP
- Effect sizes that might help inform sample-size estimates for a full trial
- Randomisation procedures and participants’ willingness to enter a deferred entry trial
- The suitability of a twelve-week deferral period and a nine-month process evaluation
- Suitability and timing of survey instruments and outcome measures
- Time needed to collect and analyse data
- Estimated resource use and costs of delivering ACP and methods for assessing costs, benefits and cost-effectiveness

## Methods

### Patient and public involvement

At the development stage of the trial, we met with patient representatives from a local charity, the Northern Ireland Kidney Patient Association, to discuss the acceptability of ACP, strategies for implementation, and the suitability of ACP and trial documentation. The steering group also included a patient with ESKD, who was invited to co-design the trial processes.

### Trial design

A deferred entry [25, 26], parallel group randomised controlled feasibility trial in which patients and their nominated surrogate were allocated in a 1:1 ratio to either immediate or deferred entry groups. Participants in the deferred entry group had outcomes measured contemporaneously with the immediate entry group but received the intervention 12 weeks after baseline data collection. This allowed a comparison at 12 weeks between those who had received the intervention at the beginning of the trial (the immediate group) and those who had not yet received it (the deferred group), as in a standard randomised controlled trial (RCT).

### Participants and settings

Participants were recruited from two haemodialysis units in Northern Ireland (NI), UK. Patients were eligible for the trial if they were English speaking; aged 65 years or older; with ESKD and receiving haemodialysis, with capacity to understand, retain, and weigh the necessary information in English and communicate their decisions; identified by their consultant nephrologist as having either worsening symptoms, functional decline, or two or more co-morbidities; and not expected to die in the next 3 months. Surrogates (a relative or friend identified by the patient as their nominated surrogate and willing to represent the patient’s wishes should they lose decision-making capacity, although without formal power of attorney) were eligible if they were aged 18 years or older and able to read, write, and speak English.

The sampling frame was patients meeting initial criteria (aged 65 or older and receiving haemodialysis) registered with the participating units. Patients on this list were screened against the full eligibility criteria by their nephrologist and a nurse trained as an ACP facilitator. Eligible patients were approached by their nephrologist to assess their interest in participating. Interested patients were given patient information packs for themselves and a surrogate. The ACP nurse returned to the patient 2–7 days later to seek their consent. Consenting patients were asked if they would like support from an ‘expert patient’ and to involve a surrogate. Surrogates could contact the research team directly using a dedicated phone number or email address, or a form and reply-paid envelope. Participating surrogates were asked to avoid discussing the details of the ACP with the patient until the research started. Surrogates could be involved with the ACP but not in the research if they preferred.

### Intervention

The intervention was offered in the outpatient units when the patient attended for haemodialysis. The lead professionals were seven experienced nurses (with 20–45 years as registered nurses, all with post-registration courses in renal nursing, and four with counselling qualifications) working in the two haemodialysis units, who had volunteered for half-day training as ACP facilitators offered by a local health and social care trust using ‘Sage & Thyme’ Communication Skills Training on dealing with people in distress [27]. Patients were given the opportunity to receive support from ‘expert patients’ during the ACP process. These were five people with ESKD who were in receipt of a kidney transplant or on dialysis and who volunteered for a 2 hour training session delivered by the team doing the ACP training, focused on understanding ACP and the role of the expert patient. The expert patient was available to act as a conversation partner with patients making an ACP, with support from an identified ACP nurse. A senior nurse in each unit, supported by a designated consultant nephrologist, oversaw the intervention process. Before the trial began, patients attending for dialysis were handed a flyer with a brief description of the research telling them that their doctor or nurse might approach them about this in the following weeks.

1. Participants were offered the opportunity to complete a plan by an ACP nurse, who discussed the process with them using the booklet, “Your life and your choices: plan ahead,” produced by the NI Public Health Agency and Macmillan Cancer Support. At this stage, the ACP nurse asked the participant to complete the ‘Record of my wishes’ form found in the booklet, intended to help patients organise their thoughts.
2. One-to-two weeks later, the patient completed an ACP document (Advance Care Planning Summary) with the help of the ACP nurse, and a trained expert patient who (if the patient wished) provided peer support at the time of ACP completion and subsequently by telephone, assisted where necessary by the ACP nurse.
3. The patient’s surrogate was invited to take part in the discussion if the patient wished.
4. The ACP document was one recommended for use throughout NI (“A record of my wishes”, developed by the NI Palliative and End of Life Care Implementation Group), which records an Advance Care Planning Summary, alongside the identification of a nominated surrogate decision-maker. The plan set out:
  - What the patient would like to happen in the future
  - What the patient would not want to happen
  - If it was already made: a record of the presence and broad content of an advance decision to refuse treatment (ADRT): a legally binding statement that allows a patient to refuse specific medical treatments if they lose capacity [13]; and any do not resuscitate (DNR) decision document.
  - Preferred place of care at the end-of-life
  - Any special requests

Patients were encouraged to keep the ACP with them and to make it available to anyone caring for them. A summary was kept with their medical notes and copied to their General Practitioner (primary care physician), and community nursing services. The ACP was reviewed with the ACP nurse after two and 12 weeks, or if circumstances changed, or the patient changed their mind about preferences. Participants in the deferred entry group were offered the intervention 12 weeks after baseline data collection, following collection of their 12-week outcome data.

### Outcomes

Outcomes were collected in the same way in the immediate entry and deferred entry groups. All outcomes were measured by trained research assistants who were aware of group allocation (Table 1).

i. Quality of life as measured by the Kidney Disease Quality of Life instrument – Short Form (KDQOL-36™), a 36-item Likert-type scale where each item is scored on a range of 0–100 and higher scores reflect better quality of life [28].
ii. Degree of cognitive impairment as measured by the Isaacs Set Test (IST 15) [29].
iii. Degree of anxiety, depression, well-being, functioning and risk as measured by the Clinical Outcomes in Routine Evaluation measure (CORE 34) a 34-item Likert-type scale scored on a 5-point scale ranging from 0 (not at all) to 4 (most or all the time). Higher scores indicate greater distress [30]. Mean scores from reference groups are 1.86 (SD 0.75) for those referred to psychiatric services and 0.76 (SD 0.59) for the general population [31].
iv. Degree to which the patient felt that they had shared in decision-making about their care as measured by the Patient Experience of Shared Decision Making (SHARED) instrument [32], a ten-item Likert-type scale ranging from ‘Disagree strongly’ to ‘Agree strongly’. Scores are out of 20, with one point for ‘Agree’, two for ‘Agree strongly’, and no points for disagreement. Higher scores indicate more sharing.
v. Agreement between the patient and surrogate in terms of the patient’s preferences, measured by asking the carer (by telephone if they were not present) to make an independent assessment of the patient’s preferences in relation to the key information covered by the ACP intervention (a-e above), before taking part in the ACP and at two and 12 weeks post ACP.
vi. An economic evaluation of costs and benefits of the ACP intervention using data from the following sources:
  - Electronic hospital admissions data for each patient
  - Patient completed cost diaries documenting personal health and social care resource use. These were paper diaries to be filled in weekly with printed sections for the patient – with help from a research assistant if required – to provide details of formal care (e.g. from a home care worker) or visits from health care professionals received in the home; contacts with community doctors or nurses; use of hospital or residential services. Patients were asked to record the number and estimated length of contacts.
  - Health-related quality of life using the SF-6D utility measure, derived from the SF-12 (contained within the KDQOL-36™ questionnaire).
  - Staff training costs.
  - Staff intervention time for each patient, documented by staff delivering ACP.

**Table 1:** Schedule of trial interventions and assessments

Time points	Immediate entry		Deferred entry	
	Patient^a^	Surrogate	Patient^a^	Surrogate
**T1** **Baseline**	IST 15 CORE 34 KDQOL-36™ SHARED	ACP agreement questionnaire	IST 15 CORE 34 KDQOL-36™ SHARED	
	ACP intervention	ACP intervention		
**T2** **2/52**	ACP 1st review^b^ CORE 34 SHARED	ACP agreement questionnaire *before* review	CORE 34 SHARED	
**T3** **12/52**	ACP 2nd review^b^ CORE 34 KDQOL-36™ SHARED	ACP agreement questionnaire *after* review	CORE 34 KDQOL-36™ SHARED	ACP agreement questionnaire
			ACP intervention	ACP intervention
**T4** **24/52**			ACP review^b^ CORE 34 KDQOL-36™ SHARED	ACP agreement questionnaire *after* review

*IST 15* Isaacs Set Test, *CORE 34* Clinical Outcomes in Routine Evaluation measure, *KDQOL-36™* Kidney Disease Quality of Life instrument – Short Form, *SHARED* Patient Experience of Shared Decision Making instrument

^a^Patients in both groups also completed a cost diary during intervention period as part of the economic evaluation

^b^Surrogate may not be at the review of the ACP with the patient (this is at the patient and/or surrogate’s discretion)

### Sample size

We aimed to recruit 40 patient-surrogate dyads. Assuming 25% attrition, this sample size provided sufficient numbers to allow feasibility to be estimated [33, 34].

### Randomisation

The random allocation sequence was generated by a statistician unconnected with recruitment, using randomisation software, into blocks of random sizes from two to eight. Block sizes were concealed from the clinical staff involved in recruitment. Allocations were concealed using sequentially numbered, opaque, sealed envelopes, held by a research team member unconnected with recruitment or data collection. These envelopes were opened sequentially following patient consent and baseline data collection.

### Blinding

Analysis of outcome data was carried out by a statistician and health economists blinded to allocation. However, it was not possible to blind patients, their surrogates, or clinical staff to the ACP intervention. Neither was it feasible in a study with limited resources to blind outcome assessment.

### Analytical methods

Baseline data were analysed using descriptive statistics. The proportion of patients eligible for inclusion, agreeing to participate and completing the study was calculated along with exact binomial 95% confidence intervals (CIs). Analysis of covariance was used to compare the mean difference (and 95% CIs) in outcome variables at 12 weeks between the intervention and control group adjusting for baseline values [35]. Paired t-tests were conducted to compare changes in outcome measures within the immediate entry and deferred entry groups. Statistical analyses were conducted using STATA SE software, v14.0 [36].

### Economic analysis

A cost-consequence analysis was undertaken alongside the randomised trial.

We tabulated the mean per patient volume of health-system resource use (healthcare activity) and mean costs per patient. The difference in costs between groups at 12 weeks was calculated. Standard deviations and 95% CI were used to describe precision estimates. Unit costs were informed by NHS 2017/18 reference costs and market rates. Costs are presented in 2018 Great Britain Pounds sterling (£) and discounting was not applied.

The mean preference-based quality of life (utility) for ea13-ch group was calculated at 12 weeks, using UK tariffs for the SF-6D, and adjusted for baseline values. Missing data were clearly reported, and complete case analysis used in the first instance. To assess uncertainty in utility estimates, a sensitivity analysis imputing the last value carried forward (if appropriate) was undertaken. Quality adjusted life years (QALYs) were calculated in a similar fashion for each group. STATA SE software (v14.0) was used for the economic analysis and reporting was consistent with the Consolidated Health Economic Evaluation Reporting Standards (CHEERS) checklist [37]. As it is unclear whether a cost per QALY framework is appropriate for decision making about interventions near the end of life, costs and outcomes were not aggregated into a cost-effectiveness ratio for this feasibility study.

### Process Evaluation

The process evaluation was informed by the findings of a realist review on implementation of ACP with patients with ESKD [23]. In relation to the trial, this was a mixed-methods sequential explanatory approach in which the qualitative data are collected after the quantitative data to help explain how the quantitative results have come about [38]. Research assistants observed staff training for ACP. They also developed a process map [39] of the personnel and systems involved in managing ACP with help from key nursing and medical staff who had delivered the intervention from the unit recruiting the majority of participants. Research assistants met with medical and nursing staff who had delivered the intervention. These staff described in detail their real-world experience of implementation, outlining all the necessary steps for delivering ACP in practice. Qualitative data was collected from those delivering and making ACP, using a semi-structured guide, to elicit their experience of ACP (what they hoped ACP would achieve; their concerns and feelings) and their views on barriers and facilitators of implementation (the written materials used; what helped or hindered; what could be done better). We convened focus groups with four “Sage and Thyme” ACP trainers, and three ACP nurses. We also interviewed four nephrologists, six patients and five surrogates. Interviews and focus groups were digitally recorded and transcribed verbatim. Each piece of interview and other data was coded in relation to the initial theory derived from the realist review to allow indexing and retrieval in a suitable database. The qualitative data were reviewed by members of the research team searching for configurations that support, contradict and link theory, seeking to explain outcomes.

### Ethical approval

Ethical approval was provided by the Office for Research Ethics Committees, Northern Ireland. REC reference: 16/NI/0043; Protocol number: B16/06; IRAS project ID: 193402. All participants gave written informed consent. ClinicalTrials.gov Identifier: NCT02631200. The trial adheres to CONSORT guidelines for randomised pilot and feasibility trials [40].

## Results

### Recruitment and time needed to collect and analyse data

**Fig. 1:**
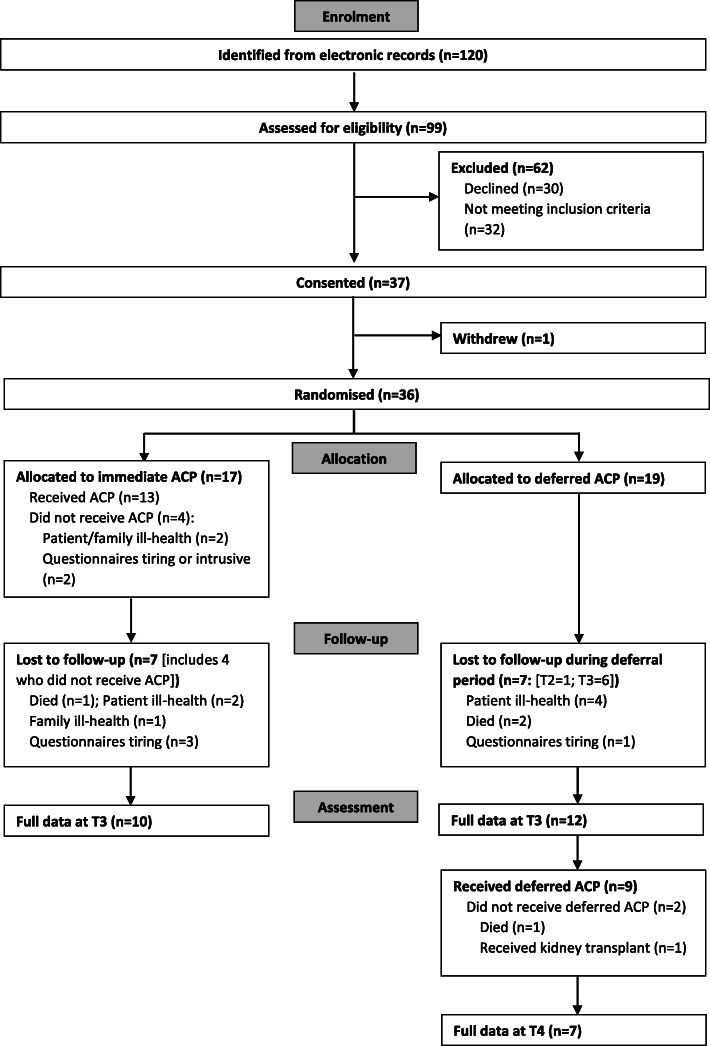
Flow of participants through the trial

The research took place between December 2016 and August 2018. Recruitment lasted 189 days, with an intervention and data collection period of 443 days. Patients identified from electronic records as meeting initial recruitment criteria numbered 120. Of these, 99 were screened against the full eligibility criteria by their nephrologist and a nurse trained as an ACP facilitator, and 32/99 were excluded: seven lacked mental capacity; six were sight or hearing impaired; six had a recent acute illness or decline; four had less than 3 months life expectancy; two had not experienced either worsening symptoms, functional decline, or two or more co-morbidities; one had limited English, and for six patients no reason was given. Sixty-seven of the 99 patients (68, 95% CI 58–77%) were invited to participate and 30 (45%) declined. Of the 24 patients who gave a reason for non-participation, 11 (46%) objected to ACP; four (17%) did not want to distress their spouse by raising ACP; three (13%) did not want the trouble of answering questions; one did not want to choose a surrogate; and the remaining five did not disclose specific reasons. Thirty-seven patients consented to join the trial, with one patient withdrawing before baseline data collection at T1, leaving 36 (54% of those invited, 95% CI 41–66%) to be randomised: 17 to the immediate intervention group and 19 to the deferred intervention group. The planned sample size was almost reached (Fig. 1).

### Retention and participation

Of all 36 participants, 22 (61, 95% CI 44–77%) made an ACP and completed data collection at T3. In the immediate entry group, 13 received the intervention and completed data collection at T2 (2 weeks); with 10 completing data collection at T3 (12 weeks). In the deferred group, 18 entered the follow-up period and completed data collection at T2; with 12 completing data collection at T3, 9 receiving deferred ACP, and 7 completing data collection at T4 (24 weeks). Only one patient chose to involve an expert patient. By T4, 17 patients (47%) remained in the trial and 19 (53%) had left, 12 of these from the deferred group. Four of these 19 patients had died; six felt physically unable to complete the questionnaires; one received a transplant; one felt too anxious about the research process; one left because of family ill health; and six did not specify a reason for leaving.

Surrogate involvement was low, with 17 of 36 patients (47.2%) able to formally identify a surrogate willing to be named in the ACP document, and 10 surrogates fully participating in the ACP process and providing information on their understanding of the patient’s preferences (Table 2).

**Table 2:** Surrogate participation

	Immediate	Deferred	All participants
Patients	17	19	36 (100%)
Surrogate named in ACP document	10	7	17 (47.2%)
Participated in ACP process	7	3	10 (27.8%)
Did not participate in ACP	3	4	7 (19.4%)
Converged^a^	3	0	3 (8.3%)
No convergence	4	3	7 (19.4%)

^a^Converged = surrogate’s understanding of ACP converged with patient’s on at least one dimension of ACP

### Baseline data

Baseline data are presented in Table 3. The groups were broadly similar with minor imbalances consistent with randomisation of a small number of participants. All participants were white British or Irish.

**Table 3:** Baseline characteristics

Description	Immediate entry (***n*** = 17)	Deferred entry (***n*** = 19)	All (***n*** = 36)
Age (years)^a^	75.6 (6.81)	73.2 (5.01)	74.9 (6.89)
Age range (years)	67–92	65–83	
Sex (male)	14 (82%)	16 (84%)	
RRT ^b^ (years)^c^	1 (0.5–4.0)	3 (1.0–7.0)	2 (1.0–5.5)
Number of comorbidities^c^	2 (1.0–4.5)	3 (2.0–4.0)	2 (1.0–4.0)
Diabetes	7 (41%)	5 (26%)	
Isaac’s Set Test^a^	31.7 (7.4)	30.6 (6.3)	31.1 (6.7)
CORE34^a^	0.48 (0.43)	0.75 (0.57)	0.62 (0.52)
KDQOL-36™			
Symptoms^a^	77.1 (18.6)	67.9 (23.1)	72.2 (21.3)
Effects^a^	86.8 (12.1)	65.8 (25.6)	75.7 (22.7)
Burden^a^	45.6 (27.5)	40.8 (30.2)	43.1 (28.7)
SF12 physical^a^	37.3 (4.8)	30.7 (10.46)	33.8 (8.8)
SF12 mental^a^	46.8 (10.7)	51.3 (12.4)	49.2 (11.7)
SHARED score^a^	11.5 (6.9)^e^	8.3 (4.1)^e^	9.3 (5.2)
University degree	5 (29%)	1 (5%)	
Household income^d^	£10–20,000 (£10–60,000)	£10–20,000 (£10–70,000)	
Renal Unit 1	13 (76%)	16 (84%)	

^a^Mean (standard deviation)

^b^Renal replacement therapy

^c^Median (inter-quartile range)

^d^Median, reported in increments of £10,000 (range)

^e^Data from 6 patients in immediate group and 13 in deferred group

### Outcome measures

Outcome measures are presented in Table 4. KDQOL-36™ scores were commensurate with those of older adults receiving dialysis in the United States [41]. Between T1 and T3, KDQOL-36™ scores fell in the immediate group and rose in the deferred group, and these changes were statistically significant in the Effects, Burden and SF12 physical subscales. CORE34 scores were low compared with the general population [30]. They fell in the deferred group between T1 and T3 but this change was not statistically significant.

**Table 4:** Outcome measure scores at baseline T1 and T3 (including only people who contribute to complete case analysis)

Variable	Immediate (***n*** = 10)			Deferred (***n*** = 12)				
	Baseline T1	T3	Within group P	Baseline T1	T3	Within group P	Adjusted diff in mean^**b**^ (95% CI)	ANCOVA P
KDQOL-36™								
Symptoms^a^	81.7 (17.1)	75.4 (13.3)	0.116	71.4 (20.7)	78.3 (19.2)	0.154	−9.3 (−20.7,2.1)	0.103
Effects^a^	89.1 (12.8)	78.4 (16.0)	0.024	68.0 (24.1)	73.4 (21.6)	0.084	−12.2 (−23.3,-1.1)	**0.033**
Burden^a^	56.9 (19.6)	46.9 (23.8)	0.100	41.7 (23.9)	55.2 (25.7)	0.001	−22.6 (−36.0,-9.1)	**0.002**
SF12 physical^a^	38.3 (4.7)	31.5 (9.5)	0.008	33.2 (11.2)	34.9 (13.0)	0.513	−8.4 (−15.8,-1.0)	**0.029**
SF12 mental^a^	51.7 (6.2)	53.3 (6.9)	0.608	53.1 (10.7)	52.4 (6.9)	0.816	1.2 (−5.0,7.3)	0.698
CORE34	0.4 (0.4)	0.4 (0.3)	0.293	0.6 (0.3)	0.4 (0.3)	0.01	0.2 (−0.0,0.3)	0.056
SHARED^c^	14.5 (7.8)	12.0 (5.7)	0.344	7.8 (4.0)	11.2 (6.0)	0.167	−4.5 (−17.3,8.3)	0.410

^a^Mean (standard deviation)

^b^ANCOVA adjusted for baseline values

^c^Data from 2 patients in immediate group and 6 in deferred group

Many patients found the SHARED tool difficult to use because the baseline measure required them to think of a time when they had made a decision with a healthcare professional, and few could identify such an occasion. Consequently, few completed this measure.

### Cost consequence analysis

Admissions data for the study period were readily available from electronic hospital records at each site. Patients were able to provide information for the cost diaries but most did not record data at home and instead waited until attending dialysis to report this information to research assistants. Initially, we recorded these data weekly but we changed to monthly because so few events were reported. Clinical staff recorded their time in minutes spent at each stage of the research and for the ACP implementation.

Resource use was categorised into ACP intervention costs: nephrologist and nursing staff time for training and implementation, and expert patient facilitation time (Table 5); and health system costs: hospital admissions, formal home care, GP visits, and residential aged care services (Table 6). The mean time taken by nephrologists to introduce ACP was 16 min, and nurses 19 min. The mean nursing time taken to deliver the ACP was 74 min. Following randomisation, the average length of stay for those admitted to hospital over 12 weeks was 10 days for the immediate group and 9 days for the deferred group. There was no statistically significant difference in health system resource use or costs between the immediate and deferred group for any cost category.

**Table 5:** Staff costs to deliver ACP

Resource use category	Staff time in minutes (SD)	Mean staff costs (SD)	Training fees
Nephrologist introducing ACP per patient – completing patients ^a^	16.3 (10.4)	**£**29.34 (18.72)^i^	
Nephrologist introducing ACP per patient – withdrawing patients ^b^	13.8 (8.0)	**£**24.84 (14.4)^i^	
Nurse introducing ACP per patient – completing patients ^c^	19.0 (9.4)	**£**14.30 (7.08)^j^	
Nurse introducing ACP per patient – withdrawing patients ^d^	13.8 (9.6)	**£**10.38 (7.23)j	
Nurse delivering ACP per patient ^e^	73.5 (15)	**£**55.33 (11.29)j	
Training time per staff participant ^f^	270	**£**365.26	**£**80 (*n* = 10)
Training time per expert patient ^g^	120	**£**100	**£**40 (*n* = 5)
Compensation per expert patient ^h^		**£**50	

^a^data from 11 patients

^b^data from 12 patients

^c^data from 17 patients

^d^data from 20 patients

^e^data from 13 patients (9 immediate and 4 deferred ACP)

^f^3 nephrologists and 7 nurses

^g^5 expert patients

^h^1 expert patient

^i^Cost per hour, 48-h week of a hospital based medical consultant - **£**108

^j^Weighted average of hourly rate of hospital based nurses. ACP Nurse pay bands: one band 7, four band 6, and one band 5 (one nurse was trained but did not implement ACP) [48]

**Table 6:** Health system resource use and costs

Resource use category	Immediate (***n*** = 13)		Deferred (***n*** = 13)		Difference in resource use		Immediate £ (***n*** = 13)		Deferred £ (***n*** = 13)		Difference in costs£	
	Mean	SD	Mean	SD	Mean	95%CI	Mean	SD	Mean	SD	Mean	95%CI
Hospital admissions (length of stay)	10.46	14.05	9.08	10.37	1.38	−8.61 to 11.38	16,769	22,514	14,550	16,626	2219	−13,801 to 18,240
GP practice or GP out of hours service	2.69	4.33	3.92	4.50	−1.23	−4.80 to 2.34	24.47	54.22	36.37	48.29	−11.91	−53.47 to 29.66
Formal home care	13.08	39.89	6.46	11.02	6.62	−17.07 to 30.30	87.54	238.6	118	244.5	−30.46	− 226 to 165.10
Residential services	0	0	0.23	0.6	−0.23	−0.59 to 0.13	0	0	41.25	102.56	–	–
Total							16,881	22,586	14,742	16,639	2139	−13,919 to 18,198

The mean quality of life (utility) for the immediate intervention group decreased by 0.01 at 12 weeks, while it increased by 0.03 for the deferred group. However, neither change was statistically significant and there was no significant difference in utility between groups at 12 weeks when adjusted for baseline values (Table 7). A similar result was observed for QALYs.

**Table 7:** Preference-based quality of life scores (utilities) and quality adjusted life years (QALYs)

SF-6D utility	Immediate (***n*** = 13)				Deferred (***n*** = 13)					
	Baseline (T1)		12 weeks (T3)		Baseline (T1)		12 weeks (T3)		Difference between groups	
	Mean	SD	Mean	SD	Mean	SD	Mean	SD	Mean^a^	95%CI
	0.69	0.09	0.68	0.11	0.68	0.15	0.71	0.13	−0.05	−0.15 to 0.05
QALYs	**Immediate (n = 13)**				**Deferred (n = 13)**					
	12 weeks (T3)				12 weeks (T3)				Difference between groups	
	Mean		SD		Mean		SD		Mean	95%CI
	0.12		0.07		0.13		0.07		−0.005	−0.06 to 0.06

^a^Difference between groups adjusted for baseline values

### ACP content

Data were available for 17 patients and their surrogates. No patients reported having made an advance decision to refuse treatment or a do not resuscitate document. Thirteen of 15 patients identified home as their preferred place of care at the end-of-life. In terms of their preferences for future treatment, patients were able to speak in broad terms (for example, that they would like to continue with dialysis; or that they would not want to be kept alive in a vegetative state; or that they would wish treatment to continue even if quality of life was affected) but many were unable to articulate preferences in specific hypothetical circumstances. Eight patients were content to trust their family in conjunction with the medical staff to take decisions in their best interests. Special requests centred on funeral and religious arrangements.

Agreement between the patient and their surrogate in terms of the patient’s ACP preferences is presented in Table 2. We included patients and surrogates who made an ACP from both immediate and deferred groups. We considered the surrogate to have converged if their understanding moved towards the patient’s views on at least one dimension of ACP. Overall, only ten surrogates participated fully in ACP. Of these, three converged and seven did not.

## Process evaluation

This section begins with an overview of ACP implementation as described through process mapping, then provides detail on the experiences of patients, surrogates and staff. Trainers are identified as T1, T2 etc.; patients as P1, P2; surrogates S1, S2; consultant nephrologists CN1, CN2; and ACP nurses ACPN1, ACPN2 etc.

### Process mapping

**Fig. 2:**
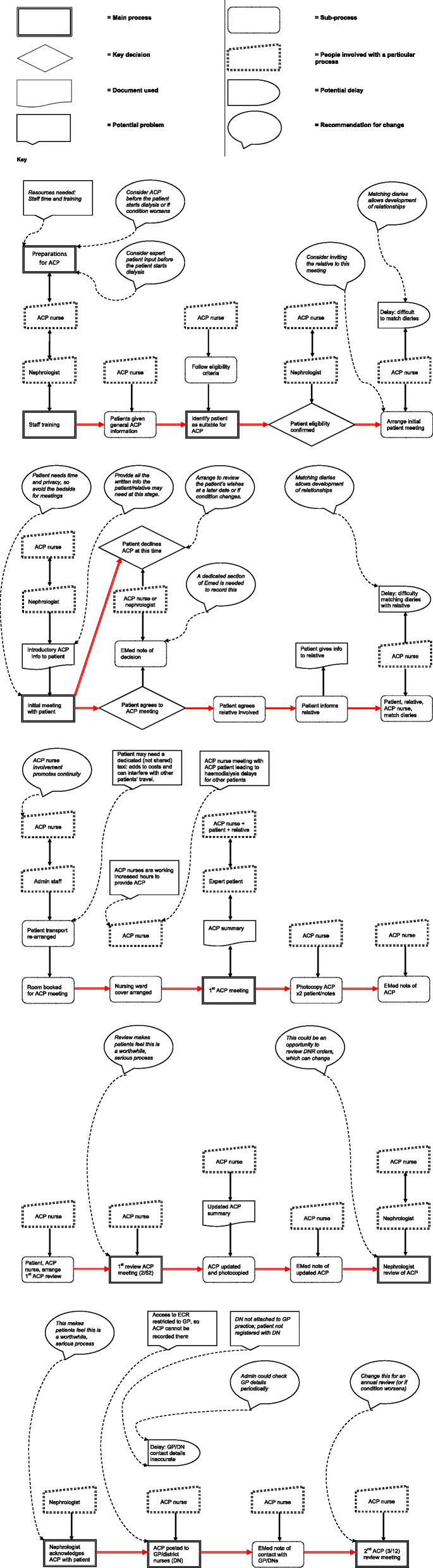
Process map: Advance care planning, Haemodialysis Unit

This mapped ACP implementation from staff training through to the second ACP review. It helped reveal the complexity and challenge of ACP implementation, with each plan comprising 10 major and 15 minor processes, about a third of which were patient contacts. ACP nurses were associated with 21 of those processes and nephrologists with six (Fig. 2).

### Staff training for ACP

Both doctors and nurses took part in half-day training for ACP with a multidisciplinary group of experienced, well-prepared trainers. The trainers expressed a thoughtful and nuanced view of the nature of ACP, embracing both practical and relational aspects of ACP. Having multidisciplinary representation from across a renal team that was already working together was thought to enhance the training.

‘*And I know everybody kind of really, kind of knew each other. But you had everybody. You had consultants there. You had nurses. You had…everybody was there to learn. And everybody chatted away. They talked and gave answers. And they really motivated to get this rolling, to get to talk to their patients about advance care planning.’ (T1)*

*‘So, I think when you get a group who have a focus on and also have a structure within which they are going to apply this knowledge, I think that makes a difference. It makes the training quite different.’ (T2)*

Trainers identified challenges in terms of the suitability of the venue and staff having opportunity to attend.

*‘I suppose it’s just again that staff can actually get to it… shortages on the wards or in the community and they can’t actually come to the training.’ (T1)*

Despite the insights and preparation of trainers, attending staff reported that training related to the ACP process was too brief and too general – focused on communication about prognosis rather than preparations for delivering ACP – but the training was still helpful in that it provided an opportunity for reflection and discussion.

*‘…it felt much more like an advanced communication course…but it wasn’t about which I was hoping to be more about the nuts and bolts, the fundamentals, how do I actually set this up, how do we run it, how do we keep it going?’ (CN1)*

### Patient experiences of ACP

Interviewed patients reported positive responses to ACP and felt that it had helped them discuss and plan for their final illness.

*‘Yeah, it was fine. We’re just as we are today. I was absolutely at ease … I thought it was done all right, like, you know, I was happy enough.’ (P1)*

*‘I didn’t want to be resuscitated anymore….I wouldn’t like to spend my last days in a nursing home.’(P2)*

*I feel happy enough with it. At least I have dotted the ‘Is’ and crossed the ‘Ts’. … there was nothing unhelpful. I mean, the only thing was the withdrawing care, which I don’t agree with at all under any circumstances. ….I want to live until I die. (P3)*

ACP helped patients clarify their wishes with both staff and family, and so engendered a sense of relief.

*Well, I think it forces you to …. think about it end of life and anything surrounding that. So, it helped me concentrate that- it helped me also stimulate a conversation with my wife …. I suppose I’m, yeah, more content with where I am, and… the direction I’m going. (P4)*

*‘Probably relieved a wee bit, you know…because it made my son aware of my wishes….you’re able to discuss things a bit better, like, you know, with my family’ (P3)*

Patients professed themselves at ease with involving their relatives in ACP and found this useful to help with the discussion.

*Well, it’s fine (including a relative in making the ACP). It was fine. I mean, she and I have a great relationship…. And in fact, she had far more ideas than I had. (P3)*

### Surrogate experiences of ACP

Surrogates reported that ACP made them more aware of the patient’s wishes and stimulated family discussions, which reduced uncertainty and gave them increased confidence that they would be able to make appropriate decisions.

*‘I thought it’d probably be a good idea because it sorts things out and maybe decisions could be made that, that wouldn’t be up to me, you know, that would probably take some of the onus off me from having to make some decisions.’ (S1)*

*‘I was able to tell the children, you know, that if anything happened everything in here is for dad, I wouldn’t have probably been able to talk to them about it.’ (S2)*

This was the case even when the surrogate was not in agreement with the patient’s wishes.

*‘He said, “Oh I want to be resuscitated as many times as possible”, and I was like, “What? Why would you do that?” And he goes, “Because I do.” He says, “I got a fighting chance,” and I said, “But, you won’t get a fighting chance because you may come back not right,” and he says, “But that’s what I want to be resuscitated,” and I said, “Well your choice.” Yeah, so that was fine …I have respect for him … I mightn’t agree with what he wants but that I make sure that it is done because it’s the way he wants it.’(S3)*

### Experiences of ACP nurses and doctors

When discussing ACP with patients, a wide range of non-medical aspects were covered, such as helping patients arrange where they would like to die and formulating wills but much less discussion on the dying process or realistic medical outcomes.

*‘So I had a few patients that had been talking about writing a will but until they actually did this plan, they haven’t actually…I don’t remember any patient really focusing on the final days or hours.’ (ACPN1)*

*‘They were very unrealistic: “Oh yes, I want the full everything, treatment.” But you might not survive.’ (ACPN2)*

On the other hand, ACP could be a useful process for starting discussions.

*‘I think ACP is about beginning the dialogue with patients about what is it that they would want to happen in certain circumstances. And sometimes that’s relatively easy because it’s easy to define things like dialysis or resuscitation and things. But other things are not so straightforward…Some people say I wouldn’t want this if I had a stroke but you could have a stroke with very minimal changes or a stroke that was totally incapacitating so sometimes it’s difficult to get the details right but I think it’s very helpful in initiating conversations with people.’ (CN2)*

*‘…having a dialogue where we explore the wishes and fears of the patient…’ (CN3)*

As indicated by surrogates, ACP was thought to help relieve families of some of the burden of decision-making.

*‘… what do their loved ones think of the future? Are we all being reasonably realistic about the expectations of health for the future of that patient?… a way of broaching a difficult subject which is really just to tease out the fact that we all have finite lifespan” (CN4)*

*‘It takes the burden off the family, they’re not left in a visitor’s room having to make a decision … you have done everything that person would have wanted, so it would help with the whole grieving process rather than you’re thinking, I made that decision, and was that the right decision, you know,’ (ACPN3)*

### Personal impact on staff

Although staff were supportive of ACP, they found it personally challenging and felt that formal support should be made available.

*‘So, it is a very emotional process for people involved…it is also emotionally draining at times… [staff should be supported to] discuss their own issues they’ve had or things that have arisen during the process.’ (CN2)*

*‘…you’re writing this in the event that something may happen to you that you can’t voice your own wishes…And it suddenly made me think what I would like, what I wouldn’t like, if you’re ever in that position yourself.’ (ACPN3)*

### Factors thought to help or hinder ACP

A key issue was the timing of ACP in relation to the patients’ capacities and stage of disease. Those thought closest to death and therefore most in need of ACP were also those least able to engage with the burden of making a plan. Consequently, staff believed ACP should be offered earlier in the disease trajectory.

*‘I think I would like to see it introduced at an earlier stage because as we’ve seen as the trial has progressed, patients withdrew mainly because they were sick, and then we had a lot of deaths too during the study so I think introducing that at an earlier stage in the patients journey, it’s a better time for staff I think as well as patients.’ (ACPN1)*

Nurses and doctors collaborating effectively was thought to support a successful process.

*‘…actually it’s useful to have the consultant involved early on, and I think our uptake of the patients who were interested was slow until the consultants got involved, so I think the combination of the nurse involvement and a consultant together worked, seemed to work well, so that was a learning point for us.’ (CN4)*

It was acknowledged that ACP required a holistic and individualised approach, matching staff and resources to the particular needs of the patient.

*‘[Patients] varied you know, in terms of like patients who were here a long time, you know, years, more than eight or nine and asking patients who were maybe only starting dialysis, you know, so they’re very varied in terms of where they are…maybe [some] were more ready than others.’ (ACPN1)*

*‘…tailoring written information and tailoring verbal information is so important…some are comfortable reading, some are less comfortable reading…giving written information isn’t a substitute for making sure that people actually fully understand what they’re participating in.’ (CN3)*

A renal healthcare culture focused on treatment and cure, together with a societal culture of avoiding discussion of death was thought to hinder staff broaching prognosis, quality of life issues, and end-of-life care.

*‘[Patients are] not always aware that it’s a life-limiting illness and they do need to think about the future.’ (ACPN1)*

*‘I don’t think it’s an area that has got a narrative around it, it’s not something that people have been talking about, it’s not part of docu-dramas or it’s not on soaps about making advance care plans, so it’s a reasonably foreign concept to patients…concerns perhaps about the legal basis of it, people are maybe a wee bit shy…’ (CN3)*

Organisational limitations in terms of lack of dedicated time and facilities were identified as key hindrances to ACP. In addition the communication infrastructure for ACP to be disseminated to community healthcare services was a concern.

*‘…in terms of coming back to implementing it, into routine practice, it takes time, it takes time to do it well. It requires recurrent review because the patients are going to change, their prognosis is going to change, their feelings are going to change, their wishes are going to change and you need to be able to come back to that.’ (CN3)*

*‘The biggest issue we found was that of time and finding an appropriate place … to do it … it’s a very time-consuming thing in trying to fit it into normal practice…actually relied on us just doing it on our own time essentially.’ (CN2)*

*‘I just would want to make a comment about the dissemination of the plans which is not as straightforward as maybe people thought it would be. And that for the GPs are sent a copy, but the GPs got it recorded in a system that will not necessarily be available for everybody in the community to access.’ (ACPN1)*

### The burdens and benefits of implementing ACP as part of a trial

Doing ACP in the context of a clinical trial had both benefits and burdens for clinical staff.

*‘Because we’re nurses, and then a time factor as well, maybe come out of your break earlier, left break earlier to try and catch up with the documentation, so it was a time factor.’ (ACPN3)*

*‘...there seemed to be pages upon pages [of trial documentation]… (ACPN1)*

*‘…actually the study made it helpful because you could tell patients that this was part of a study, that it wasn’t that you had any particular concerns about them. And it’s very difficult to gauge end of life in renal failure patients so it was a nice way to begin the conversation.’ (CN2)*

## Discussion

Our study showed that recruitment of patients receiving haemodialysis and their surrogate decision makers into a trial of ACP is possible but not without its challenges. Recruitment was planned to take 3 months but took just over 6 months. Time needed for recruitment could have been shortened by more intensive deployment of clinical staff but this has resource implications. About a third of patients assessed for eligibility were excluded, and nearly half of those invited declined to take part. We had anticipated 25% attrition but it reached 39% at 12 weeks and 53% at 24 weeks. This suggests that planning the sample size for a full trial would require the pool of potentially eligible patients to be at least three times larger than the number required for initial participation, and five times larger than the number required to complete data collection at 3 months.

The challenges of recruitment to trials of palliative care (Boland et al., 2015) and ACP are well known (Sullivan, Garner and Dubbert, 2016; Stewart et al., 2018). Our ratio of recruitment was consistent with that of one small (Song et al., 2010) and one large trial (Song et al., 2015) of ACP among dialysis patients in the USA, but our figures for attrition following consent were higher than in the larger trial, which reached 30% at 2 months. This may be because our eligibility criteria meant we predominantly recruited frailer patients, and indeed, participating clinicians recommended approaching patients earlier in their disease trajectory.

Our study was designed as a deferred entry trial. A deferred entry trial has the advantages of a standard RCT because outcome data is collected from the deferred group before they receive the intervention, so allowing a comparison at that stage between those who have received the intervention and those who have not. It is also thought to encourage participation and reduce distress because all participants will eventually receive the intervention. A limitation is that this constrains the time at which the outcome data is collected as this is also the period that deferred patients must wait [25]. In our case, neither research assistants nor ACP nurses, nor interview data from participants reported patients being distressed by the offer of ACP, or by having to wait for the intervention, suggesting that a deferred entry trial may be unnecessary. The dialysis unit appears to lend itself to the ACP process in that it provides the opportunity for the development of supportive relationships with staff and regular contact to make an ACP.

Our sample was too small to be able to show differences in outcome measures between the immediate and deferred groups. Research assistants reported that patients were not distressed by the content of the data collection instruments but found the volume of data collected burdensome. In terms of the timing and frequency of outcome measures, there were minimal differences between scores at baseline and T2 (2 weeks), suggesting that these data do not add anything useful. Outcome measurement at three and 6 months post intervention may be more likely to capture immediate and longer-term differences between groups. The SHARED tool proved unsuitable in itself, largely because patients were inexperienced in shared decision-making, suggesting that the ACP intervention needs to include support for developing these skills and that other measures should be tried to assess shared decision-making with this population.

Detailed data on staff time spent on ACP related tasks were collected successfully along with the documentation of the ACP process. This estimate is probably conservative, as we did not anticipate all of the sub-processes of the intervention. Data on hospital admissions were readily available from routinely collected administrative data.

Fifteen patients – 22% of those approached - refused to take part in the trial because they objected to ACP or were concerned that it would distress their spouse, perhaps reflecting continued misgivings and misunderstandings about ACP in the UK. Less than half the patients were able to involve a surrogate and there was a very small effect on convergence of views on ACP, perhaps because much ACP content was at a level of broad, non-specific preferences. This may partly be due to limitations in our intervention, which provided an opportunity for the patient and surrogate to discuss and plan future preferences but evidently did not succeed in helping patients to fully clarify their values or consider specific treatment choices. It may also be due to the inherent difficulty patients experience in imagining both possible medical conditions and decisions about complex major treatments they have not yet experienced [42]. Nevertheless, those who did take part were content to make an ACP, with both patients and surrogates feeling relieved to have done so and reporting reduced anxiety and – for surrogates - increased confidence that they could represent the patient’s wishes, with some patients saying they would trust family to work with medical staff to take decisions in their best interests. This may point to focusing ACP interventions on preparation rather than prediction, preparing patients and surrogates to participate with clinicians in making the best possible in-the-moment medical decisions in light of the patient’s values and goals. Traditional ACP outcomes may need to be supplemented with those that are defined by patients, exploring what they wish to achieve through ACP, such as starting this important conversation with family and clinical staff, clarifying values, and exploring the role of the surrogate [42, 43], as measured by instruments such as the ACP engagement survey [44].

## Conclusions

A trial of ACP and its associated costs is feasible in patients receiving haemodialysis (subject to allowance for challenges in recruitment and retention of patients and their surrogates) and could provide a reliable estimate of the effects of ACP and whether this is a good use of resources. Based on recruitment and retention in our study, if 1200 patients were assessed for eligibility and invited to participate in a randomised trial, this would result in 400 being randomised, with 200 (100 in intervention and control groups) available 6 months later to be analysed for differences in ACP engagement. For example, this would give 90% power at the 5% level of significance to detect a mean increase of 0.5 on a 5-point Likert scale using the 15-item Advance Care Planning Engagement Survey [45]. Given a mortality rate of 10% per year [46], 40 participants in each group would be expected to die during the 2 years after the intervention. This would give 80% power at the 5% level of significance to detect an increase in the proportion of patients whose wishes were known and respected at the time of death from 30 to 65% [47]. Shorter follow up for this outcome would require a larger sample because of the smaller proportion of deaths among the randomised patients (for example doubling the sample size would allow follow up over just 1 year). Widening eligibility criteria to include younger (under 65 years of age) and less frail patients, together with special efforts to engage and retain surrogates would probably improve recruitment and retention. There are just 700 patients being dialysed in 6 units in NI, so recruitment would need to include three or four larger dialysis units from elsewhere in the UK. This would increase the generalisability of results and allow the inclusion of more patients from ethnic minorities.

Experienced nurses and doctors were able to deliver ACP on-site to an acceptable standard after training for only half a day, and with ongoing informal support from the research team. However, those with less experience might require more extensive training and formal ongoing support.

A significant proportion (45%) of those invited to participate declined. About one third of these appeared to object to ACP in itself, with the remainder concerned about the impact on a spouse or the effort involved in answering research questions. Those who took part in the process evaluation professed themselves content with the process, excepting the involvement of expert patients. Therefore, whilst ACP seems to be acceptable to most patients in this population, as well as to participating clinical staff, issues of patient and surrogate understanding, and burden of research must still be addressed.

Further, the ACP intervention needs to be developed to include clarification of patients’ values and preparation of patients and surrogates to participate in shared decision-making with clinicians. This in turn has implications for training and support for staff, which should be focused on helping them gain confidence in the human aspects and practicalities of the ACP process. Outcome measures can be a burden for patients, so these should be focused on important process-related (identifying a surrogate, clarifying values) and patient-centred outcomes; as well as measuring preparedness for decision-making for both patient and surrogate.

## Data Availability

Deidentified data that underlie the results reported in this article are available to qualified researchers for approved scientific uses immediately following publication with no end date. Data access proposals should be directed to the corresponding author. The Trial Protocol is available on request.

## Abbreviations

ACP: Advanced care planning
ACPN: Advance care planning nurse
ADRT: Advance decision to refuse treatment
CHEERS: Consolidated Health Economic Evaluation Reporting Standards
CI: Confidence interval
CN: Consultant nephrologist
CORE 34: Clinical Outcomes in Routine Evaluation
DNR: Do not resuscitate
ESKD: End-stage kidney disease
GP: General practitioner
IST 15: Isaacs Set Test
KDQOL-36™: Kidney Disease Quality of Life instrument – Short Form
NI: Northern Ireland
P: Patient
QUALY: Quality adjusted life years
SHARED: Patient Experience of Shared Decision Making
T: Trainer
UK: United Kingdom
